# Protective Immunity against *Trichinella spiralis* Infection Induced by a Multi-Epitope Vaccine in a Murine Model 

**DOI:** 10.1371/journal.pone.0077238

**Published:** 2013-10-10

**Authors:** Yuan Gu, Junfei Wei, Jing Yang, Jingjing Huang, Xiaodi Yang, Xinping Zhu

**Affiliations:** Department of Parasitology, School of Basic Medical Sciences, Capital Medical University, Beijing, PR China; Aaron Diamond AIDS Research Center with the Rockefeller University, United States of America

## Abstract

Trichinellosis is one of the most important food-borne parasitic zoonoses throughout the world. Because infected pigs are the major source of human infections, and China is becoming the largest international producer of pork, the development of a transmission-blocking vaccine to prevent swine from being infected is urgently needed for trichinellosis control in China. Our previous studies have demonstrated that specific *Trichinella spiralis* paramyosin (*Ts*-Pmy) and *Ts*-87 antigen could provide protective immunity against *T. spiralis* infection in immunized mice. Certain protective epitopes of *Ts*-Pmy and *Ts*-87 antigen have been identified. To identify more *Ts*-Pmy protective epitopes, a new monoclonal antibody, termed 8F12, was produced against the N-terminus of *Ts*-Pmy. This antibody elicited significant protective immunity in mice against *T. spiralis* infection by passive transfer and was subsequently used to screen a random phage display peptide library to identify recognized epitopes. Seven distinct positive phage clones were identified and their displayed peptides were sequenced. Synthesized epitope peptides conjugated to keyhole limpet hemocyanin were used to immunize mice, four of which exhibited larval reduction (from 18.7% to 26.3%, respectively) in vaccinated mice in comparison to the KLH control. To increase more effective protection, the epitope 8F7 that was found to induce the highest protection in this study was combined with two other previously identified epitopes (YX1 from *Ts*-Pmy and M7 from *Ts*-87) to formulate a multi-epitope vaccine. Mice immunized with this multi-epitope vaccine experienced a 35.0% reduction in muscle larvae burden after being challenged with *T. spiralis* larvae. This protection is significantly higher than that induced by individual-epitope peptides and is associated with high levels of subclasses IgG and IgG1. These results showed that a multi-epitope vaccine induced better protective immunity than an individual epitope and provided a feasible approach for developing a safer and more effective vaccine against trichinellosis*.*

## Introduction

Trichinellosis is a major food-borne zoonosis caused by *Trichinella spiralis* (*T. spiralis*) infection. The occurrence of trichinellosis in humans is strictly related to the consumption of raw or undercooked meat contaminated with *T. spiralis* larvae [1]. The most important source of human infection worldwide is the domestic pig [2]. However, wild animals also play an important role in trichinellosis transmission in some countries. For example, some outbreaks have been sourced to wild boar meat consumption in Bulgaria [3]. Trichinellosis is also a large public health hazard in China. From 2005 to 2009, 15 outbreaks of human trichinellosis, with 1387 cases and 4 deaths, were reported in three provinces/autonomous regions of southwestern China. Twelve (85.71%) of these 15 outbreaks were caused by the consumption of raw or undercooked pork, which remains the predominant source of trichinellosis infection in China [4]. Trichinellosis is also an economic problem in porcine animal production and food safety [2]. The prevalence of *Trichinella* infection in swine slaughtered at abattoirs varied from 0% to 5.75% in five provinces/autonomous regions in China [4]. If this zoonosis is not controlled, it could pose a more serious public health problem because China is now the largest international producer of pork. Therefore, the development of a transmission-blocking vaccine against trichinellosis to prevent swine infection would make a practical contribution to disease control. 

During the past decades, there have been many reported efforts to develop vaccine against trichinellosis, including vaccines based on crude larval extracts [5], excretory-secretory (ES) products [6], DNA [7] or recombinant proteins [8,9], all of which induced partial protective immunity in animal models. However, these traditional vaccines present challenges in terms of safety, residual toxicity, means of transportation, and difficulties associated with sufficient mass production. A subunit vaccine based on protective vaccine antigen epitopes enables investigators to overcome the problems of conventional vaccines and provides a safer, more cost-effective approach to vaccine development [10]. 

Developments in immunology and molecular biology have shown that the epitope is an antigenic determinant of the antigen molecule that is recognized by the immune system, including the conformational and linear epitopes [11]. It was reported that an eight-amino acid conformational epitope induced a degree of protective immunity against *Schistosoma mansoni* infection that was comparable to the immunity induced by an intact protective antigen [12]. Mice immunized with a subunit vaccine derived from the epitope of hookworm vaccine antigen hemoglobinase produced neutralizing antibodies that inhibited the enzymatic activity of the parental antigen [13]. An epitope-based subunit vaccine also makes it possible to construct a multivalent vaccine by combining protective epitopes from several protective antigens [14]. Because an epitope-based vaccine induces an immune response only to the highly antigenic epitopes, which are free from the original protein scaffold, it prevents the immune escape of the whole antigen and the antigenic competition of multiple antigens [15]. Epitope vaccines have also been suggested as a strategy for counteracting pathogen escape and the development of drug resistance [16].

The life cycle of *T. spiralis* is different from other nematodes because all of its developmental stages occur in the same host. However, the nematode's antigens vary during its different developmental stages within the host [17]. Thus, it is difficult to elicit high levels of protection or sterile immunity with only a single antigen. Multi-epitope vaccines consist of different antigens from different stages, which make it possible to produce a more effective protective immune response while avoiding potentially hazardous and undesirable side effects. 

In our previous studies, two immunodominant antigens of *T. spiralis*, namely paramyosin (*Ts*-Pmy) and *Ts*-87, were cloned by immunoscreening an adult worm cDNA library with infected animal sera [9,18]. *Ts*-87 is a 38 kDa protein existing in the excretory-secretory products and on the cuticle of the adult worm [19]. Both recombinant proteins (r*Ts*-Pmy and r*Ts*-87) generated partial protection against a *T. spiralis* larval challenge in vaccinated BALB/c mice [9,20,21]. 

Additional evidence shows that paramyosin is not only the structural component of invertebrate muscle [22] but is also a functional protein that plays important roles in immune defense. Several encouraging results have demonstrated that helminth paramyosin is a good vaccine candidate. Paramyosin has been shown to induce protective immunity against *Schistosoma japonicum* [23]，*Brugia malayi* [24] and *Taenia solium* [25]. Our previous work also found that the outer membrane form of *Ts*-Pmy plays an important role in the evasion of the host complement attack [26] and RNAi-mediated silencing of paramyosin expression in *T. spiralis* results in impaired viability of the parasite [27]. These findings suggested that *Ts*-Pmy is a good target for vaccine and pharmaceutical development against trichinellosis. 

Because *Ts*-Pmy and *Ts*-87 are promising vaccine candidates, a number of *Ts*-Pmy and *Ts*-87 epitopes have been identified by screening a random peptide library with monoclonal antibodies. Partial protective immunity has been achieved by these epitopes in a murine model [28,29], which provides the basis for constructing a multi-epitope vaccine against trichinellosis. 

For this study, we produced a new monoclonal antibody (mAb), known as 8F12, that specifically recognized the recombinant N-terminus of *Ts*-Pmy (r*Ts*-Pmy-N) and induced protective immunity when passively transferred to a naive mouse. This mAb was used to screen a random peptide library, from which protective epitopes were obtained. A multi-epitope vaccine consisting of three selected epitopes from two protective proteins (*Ts*-Pmy and *Ts-*87) was formulated, and the protective effects of this multi-epitope vaccine were evaluated in BALB/c mice. 

## Materials and Methods

### Ethics statement

All experimental animals were purchased from Laboratory Animal Services Center of Capital Medical University (Beijing, China). All experimental procedures were reviewed and approved by the Capital Medical University Animal Care and Use Committee and were consistent with the NIH Guidelines for the Care and Use of Laboratory Animals.

### Parasites

*T. spiralis* (ISS 533) parasites were originally isolated from a swine source in the Heilongjiang Province of China and maintained by serial passage in female ICR mice. Muscle larvae (ML) were recovered from infected mice with a modified pepsin-hydrochloric acid digestion method as described by Gamble et al. [30]. Briefly, the infected mice were euthanized 45 days post infection. After removing the skin and viscera, the whole carcass was digested in digestion solution (1% pepsin, 1% hydrochloric acid in distilled water) at 37 °C for 4 hours. The entire digest was poured through a sieve (100 meshes per inch) into a 250 ml conical cylinder to remove the undigested scraps. The digest was allowed to settle for 40 minutes and the supernatant was then discarded. The larvae were washed three times by gravity precipitation as described and resuspended in 3-4 ml of saline with 4% gelatin. Total three aliquots of 200 µl larvae suspension after being well mixed were streaked across a slide and the number of larvae was counted under a microscope. The total number of larvae was calculated according to the average number in 200 µl suspension and the total volume. Reductions in larval burden in immunized mice were calculated according to the following formula (*a* represents mean number of larvae per gram muscle in immunized mice, *b* represents mean number of larvae per gram muscle in control mice):

% larvae reduction$\text{=}\left(1-\frac{a}{b}\right)\times 100\%$

### Production of mAb 8F12

Because the *Ts*-Pmy N-terminus (1-966bp) was recognized by the protective immune sera of *T. spiralis*-infected mice, a mAb 8F12 against r*Ts*-Pmy-N was produced by hybridoma technique. The generation of hybridomas was performed with conventional methods as described elsewhere [31]. In brief, splenocytes from a mouse immunized with r*Ts*-Pmy-N were fused with SP2/0 cells. The hybridoma culture supernatant was screened for antibodies against r*Ts*-Pmy-N by ELISA and Western blot analysis. The single hybridoma clones that produced monoclonal antibodies were obtained by limiting dilution. Hybridoma cells were inoculated into the peritoneal cavity of pristane pre-treated BALB/c mice (Sigma-Aldrich, USA) to produce ascites. The ascites fluid was collected and mAb was purified by affinity chromatography using a Protein A-Sepharose 4FF column (GE Healthcare, USA). The isotype of mAb was determined with a Mouse Monoclonal Antibody Isotyping Kit (Gibco-BRL, USA).

### Mouse antisera

*T. spiralis* infection mice sera were collected from BALB/c mice that had been infected with 400 *T. spiralis* ML for 45 days. The polyclonal antibodies of *T. spiralis* infection mice sera were purified by affinity chromatography using a Protein A-Sepharose 4FF column (GE Healthcare, USA).

### Passive transfer of mAb 8F12 and challenge experiment

The selected mAb 8F12, which acted against r*Ts*-Pmy-N, was used for a passive transfer into naive mice to test its protection against a *T. spiralis* larval challenge. Mice were divided into three groups with six mice each and three independent experiments were carried out. Within the mAb group, each mouse was intravenously injected with 500 μg of purified mAb for a total volume of 0.1 ml in phosphate-buffered saline (PBS). Two other groups of mice received the same volume of either 500 μg of polyclonal antibodies of *T. spiralis* infection mice sera or PBS only, which was the control. Each mouse was orally challenged with 400 *T. spiralis* ML at 2 hours after the injection. Mice were given the same dose of antibodies or PBS on day 4. All mice were sacrificed on day 45 post-infection. The larvae were collected from whole muscle tissue and counted. 

### Panning a random phage display peptide library

The Ph.D.-12^TM^ Phage Display Peptide Library Kit (New England Biolabs, USA) was used to screen epitopes recognized by mAb 8F12 by following the manufacturer’s instructions, with minor modifications. In brief, the wells of a 6-well microtiter plate (Costar, USA) were coated with purified mAb 8F12 (100 μg/ml) in 1.5 ml of binding buffer (0.1 M NaHCO_3_, pH 8.6) at 4°C overnight. The wells were washed 10 times with TBST (50 mM Tris, pH 7.5, 150 mM NaCl, 0.1% Tween-20), followed by blocking at 37°C for 2 h with 3 ml of blocking buffer (0.1 M NaHCO_3_ containing 5% bovine serum albumin, pH 8.6). The mAb-coated wells received 1 ml of diluted phage (approximately 1.5×10^11^ particles) and were shaken gently at room temperature for 1 h. The wells were washed as described above to remove unbound phages. Antibody-bound phages were eluted with 1 ml of elution buffer (0.2 M glycine-HCl, pH 2.2) and neutralized with 150 μl of 1 M Tris-HCl (pH 9.1). The eluted phages were titered and amplified in *E. coli* ER2738 for 4.5 h and then harvested by precipitation with PEG/NaCl (20% PEG-8000, 2.5 M NaCl w/v). The amplified phages were biopanned for another two rounds under more stringent conditions including a decreased concentration of mAb 8F12 in coating buffer, a shorter binding time and an increased concentration of Tween-20 in TBST washing buffer (up to 0.5%). After the 3^rd^ round of biopanning, the eluted phages were diluted and cultured with ER2738 on LB/IPTG/X-gal plates to select the single positive clones.

### Identification of positive phage clones

#### Enzyme-Linked Immunosorbent Assay (ELISA)

The selected positive phage clones were amplified and tested for specific binding to mAb 8F12 by indirect ELISA. Microtiter wells were coated with purified mAb 8F12 (10 μg/ml, 150 μl/well) in bicarbonate buffer at 4°C overnight and blocked with 300 μl of blocking buffer at 37°C for 2 h. Approximately 10^10^ phage particles from the selected clones were added to each well and incubated at room temperature for 1 h. The wells were washed with TBST and then incubated with 100 μl of horseradish peroxidase (HRP)-conjugated anti-M13 antibody (Pharmacia, USA) at a dilution of 1:5000 in TBS at 37°C for 1 h. The ELISA plates were developed with o-phenylenediamine dihydrochloride (OPD, Sigma-Aldrich, USA) and read at 492 nm. 

#### Nucleotide sequencing

The selected phage clones were amplified in ER2738 at 37°C for 4.5 h. Single strand phage DNA was extracted as directed and sequenced with 96 gIII primer (5’ -GCCTCATAGTTAGCGTAACG-3’, New England Biolabs, USA). The deduced amino acid sequences were aligned with the *Ts*-Pmy sequence using the MEGALIGN program of DNAStar software. 

#### Western blot analysis

To determine if the positive phages could specifically bind to mAb 8F12, approximately 10^11^ phage particles from each positive clone were transferred to a PVDF membrane (Millipore, USA) and incubated with mAb 8F12 (1:5000 dilution) in 1% (w/v) skim milk/PBST at 4°C overnight. IRDye 680LT goat anti-mouse IgG was used as the secondary antibody. An Odyssey two color infrared imaging system (Li-Cor, USA) was used to visualize the binding according to the manufacturer’s instructions. 

#### Immunoprecipitation

Two milligrams of positive phage 8F7 was incubated with either non-denaturing lysis buffer (20 mM Tris HCl pH 8, 137 mM NaCl, 10% glycerol, 1% Triton X-100, 2 mM EDTA) or denaturing lysis buffer (1% SDS，5 mM EDTA) for 30 minutes at 4°C. Protease inhibitor cocktail set II (Merck, Germany) were added fresh to the lysis buffer immediately before use. The supernatants of the lysate were incubated with 2μg of purified mAb 8G12 for 3 hours at 4°C before adding 20 μl of resuspended Protein G PLUS-Agarose (Santa Cruz Biotechnology, USA). The lysate-beads mixture was incubated at 4°C under rotary agitation overnight. The pellet was centrifuged and washed 4 times with 1.0 ml of PBS (pH 7.0), and then resuspended in 40 μl of 1x electrophoresis loading buffer. After being boiled for 3 minutes, 20 μl of supernatant was transferred to a PVDF membrane and probed with mAb 8F12 (1:5000 dilution) as described above. 

### Peptide synthesis

Seven peptides were synthesized by Aviva Systems Biology Corporation (China) with a purity of over 90% as determined by HPLC. The synthesized peptides were conjugated to bovine serum albumin (BSA) or keyhole limpet hemocyanin (KLH) by reductive amination as previously described to increase immunogenicity [32]. 

### Binding of synthesized peptides to mAb by ELISA

The wells were coated with peptide conjugated to BSA (10 μg/ml, 100 μl/well) overnight at 4°C in 0.1 M carbonate buffer (pH 9.6) and blocked with 200 μl of PBS containing 1% BSA (pH 7.2, w/v) at 37°C for 2 h. The mAb 8F12 was added (1:1000 in PBS) and incubated at 37°C for 1 h. HRP-conjugated goat anti-mouse IgG (Sigma-Aldrich, USA) was used at a dilution of 1:3000 in TBS as the secondary antibody at 37°C for 30 minutes. The color was developed with OPD and read at 492 nm.

### Immunization with individual-epitope peptides

The BALB/c mice were divided into ten groups with six animals each. Seven groups of mice were subcutaneously immunized with 50 μg each of KLH-conjugated peptide emulsified with an equal volume of ISA 50 V2 (SEPPIC, France), an water-in-oil adjuvant that stimulated strong and long lasting antibody response [33], followed by two boosts of the same dose at 2-week intervals. The other three groups of mice were immunized with the same amount of r*Ts*-Pmy-N or KLH, or PBS emulsified with an equal volume of ISA 50 V2 as the control under the same immunization schedule. r*Ts*-Pmy-N were not conjugated to KLH. Ten days after the final boost, each mouse was orally challenged with 400 infective *T. spiralis* muscle larva. Forty-five days post-infection, all mice were sacrificed and the larvae were collected from their muscles. The experiment was repeated three times. The peptide that induced the highest larva reduction was selected for a multi-epitope vaccine trial. 

### Immunization with multi-epitope vaccine

To increase protective immunity, three protective epitope peptides, including previously identified YX1 from *Ts*-Pmy [29] and M7 from *Ts*-87 [28] and the second *Ts*-Pmy epitope 8F7 identified by this study, were each conjugated to KLH and mixed to formulate a multi-epitope vaccine. BALB/c mice were divided into eight groups with six animals each. Three groups of mice were immunized with 50 μg of individual target KLH-conjugated peptide (YX1-KLH, 8F7-KLH and M7-KLH). The mice of multi-epitope vaccine group were immunized with total 50 µg of three KLH-conjugated peptide mixtures (YX1-KLH, 8F7-KLH and M7-KLH, 17 μg each). Same amount (50 μg) of recombinant proteins (r*Ts*-Pmy-N and r*Ts*-87, which were not conjugated to KLH), KLH or PBS were served as control. All the antigens (50 µg in 50 µl) including the PBS control were emulsified with an equal volume of ISA 50 V2 and administered to mice subcutaneously, followed by two boosts at 2-week intervals. Ten days after the final immunization, sera were collected from each mouse to measure antigen-specific IgG, IgG1 and IgG2a against parental peptide(s). For testing sera from mice immunized with individual-epitope vaccine, 1 µg of individual BSA-conjugated peptide was coated per well. For testing sera from mice immunized with multi-epitope vaccine, a mixture of three BSA-conjugated peptides in a total amount of 1 µg (YX1-BSA, 8F7-BSA and M7-BSA, 0.33μg each) was coated in each well. Each mouse was then orally challenged with 400 infective *T. spiralis* muscle larva. Forty-five days post-infection, all mice were sacrificed and the larvae were collected from the muscles and counted. Three independent experiments were carried out.

### Statistical analysis

**Figure 1:**
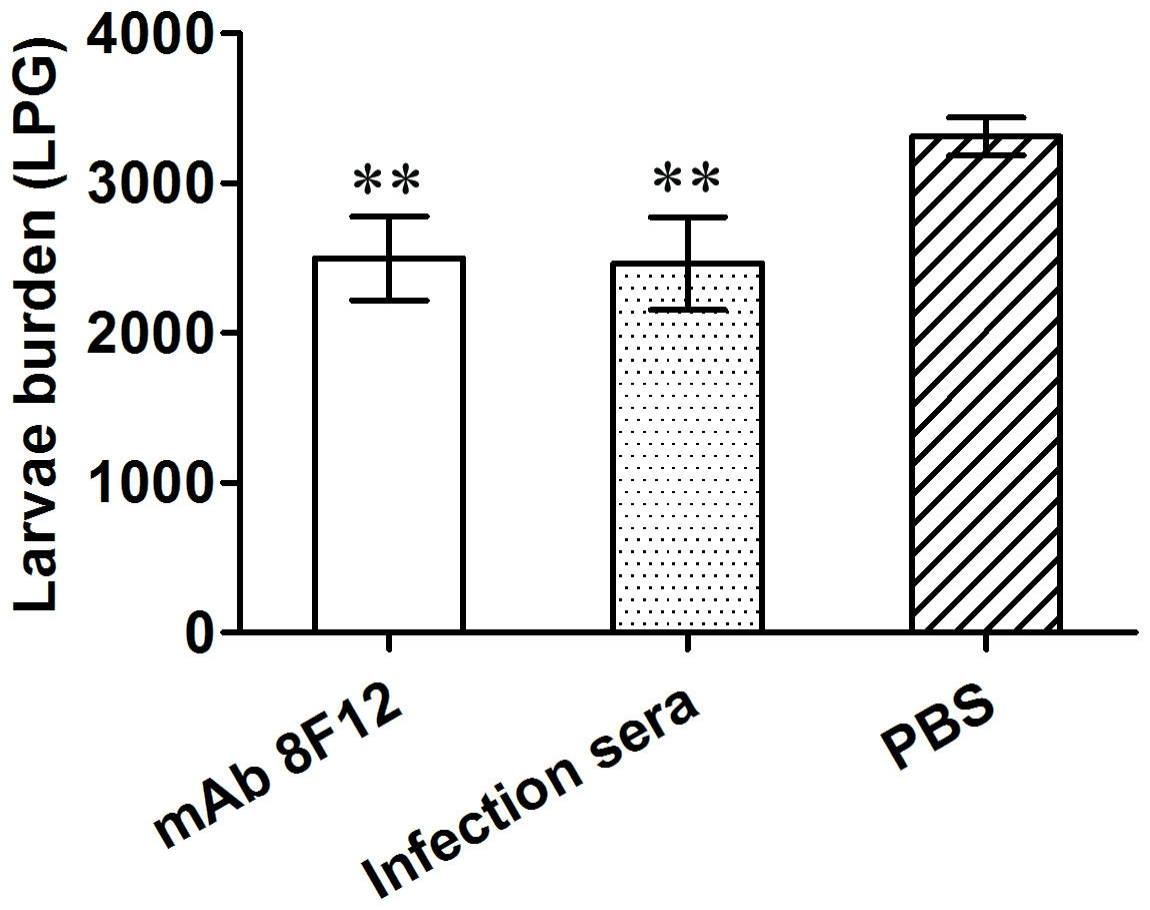
Muscle larval reduction in mice passively receiving mAb 8F12 or *T. spiralis* infection mice sera. Each mouse was challenged with 400 *T. spiralis* larvae. The challenge experiment showed that mice injected with mAb 8F12 or *T. spiralis* infection mice sera induced 24.6% and 25.6% reduction in muscle larvae burden, respectively, compared to the PBS control group (** *p*< 0.01). The larvae per gram (LPG) of muscle shown for each group represent the mean value from 6 animals with the standard deviation (one representative experiment of three independent experiments).

The data in Figures 1, 5, 6 and 7 represent the mean±standard deviation. Differences between the groups were analyzed by one-way ANOVA with the Student-Newman-Keuls method using SPSS 13.0 software. A *p* < 0.05 was regarded as statistically significant.

## Results

### MAb 8F12

 Anti-r*Ts*-Pmy-N mAb 8F12 was established by using the conventional hybridoma technique. The mAb 8F12 isotype was determined to be IgG1κ. Western blot analysis revealed that 8F12 not only recognized r*Ts*-Pmy-N (40 kDa) but also recognized the native *Ts*-Pmy (110 kDa) in crude somatic extracts of *T. spiralis* adult worms, newborn larvae (NBL) and ML (data not shown).

### Passive immunization of mAb 8F12

The protective immunity of mAb 8F12 against *T. spiralis* infection was observed following its passive transfer to naive BALB/c mice. The challenge experiment showed that mice injected with mAb 8F12 or *T. spiralis* infection mice sera induced a 24.6% and 25.6% reduction in muscle larvae burden, respectively, compared to the PBS control group (*p*< 0.01, Figure 1). There was no significant difference in the worm burden between the groups injected with mAb 8F12 and *T. spiralis* infection mice sera, suggesting that mAb 8F12 provided protective levels similar to *T. spiralis* infection mice sera.

### Biopanning and identification of positive phage clones

**Figure 2:**
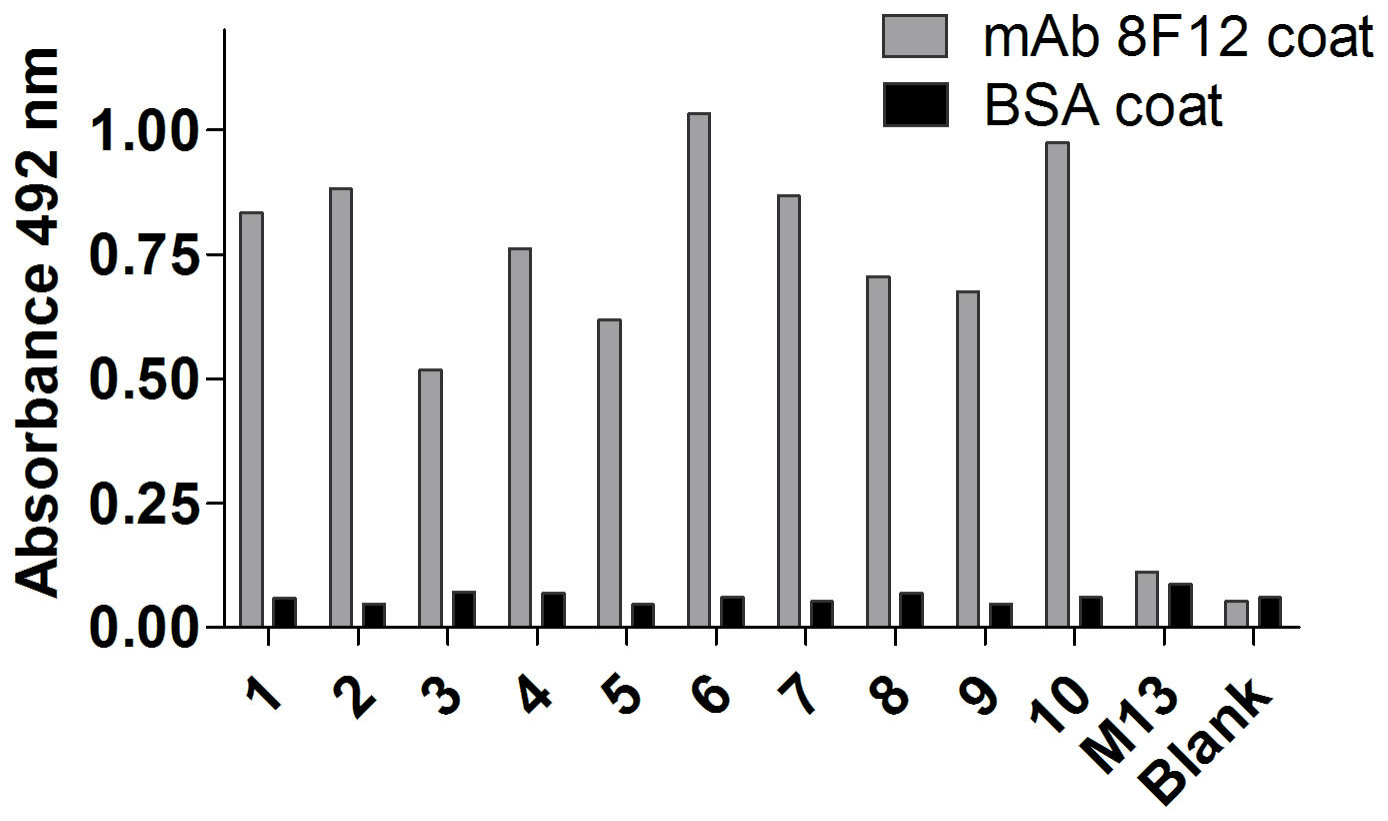
Specific binding of positive phage clones to mAb 8F12 by ELISA. Positive phage clones 1-10 showed binding activities with mAb 8F12. Wild-type M13 was used as a negative control while BSA-coated wells were used to exclude non-specific binding.

**Figure 3:**
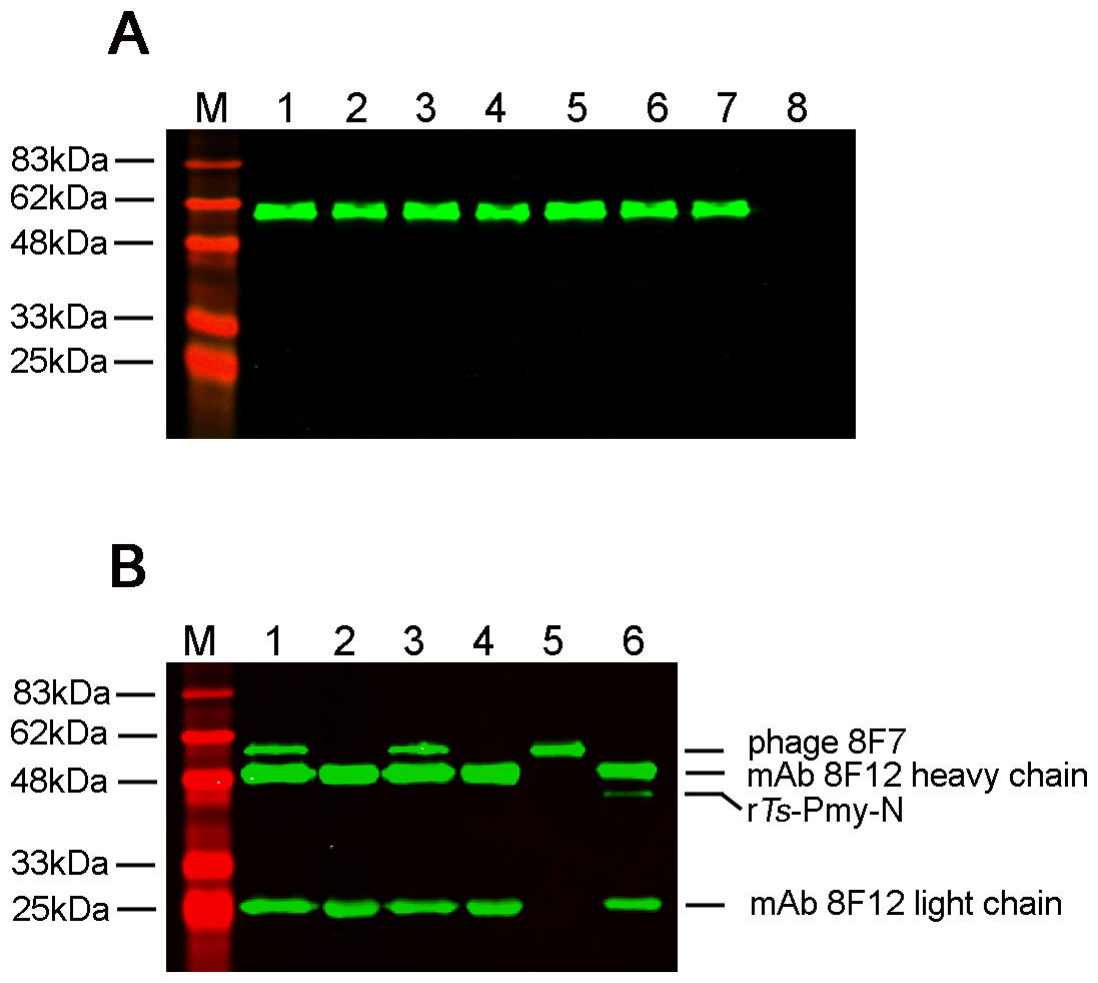
Specific binding of positive phage clones to mAb 8F12 by Western blot (A) and immunoprecipitation (B) analyses. (A). Seven positive phage clones were transferred to a PVDF membrane and a wild-type M13 phage was used as a negative control. The mAb 8F12 recognized a single band with a MW of approximately 60 kDa (displayed peptide co-expressed with phage coat protein pIII) in all seven positive clones, but not in the M13 control. M：Marker; Lanes 1-7: seven positive phage clones; Lane 8: Wild-type M13 phage. (B). Immunoprecipitation of positive phage 8F7 with mAb 8F12 at non-denatured and denatured conditions. M：Marker; Lane 1: mAb 8F12 pulled phage 8F7 in non-denaturing lysis buffer; Lane 2: mAb 8F12 pulled wild-type M13 phage in non-denaturing lysis buffer; Lane 3: mAb 8F12 pulled phage 8F7 in denaturing lysis buffer; Lane 4: mAb 8F12 pulled wild-type M13 phage in denaturing lysis buffer; Lanes 5: phage 8F7 only; Lane 6: mAb 8F12 pulled r*Ts*-Pmy-N.

Ten positive phage clones were obtained after three rounds of biopanning with mAb 8F12. ELISA results showed that each positive phage clone was able to specifically bind to mAb 8F12, but not to the BSA negative control (Figure 2). Western blotting showed that a single band with a MW of approximately 60 kDa (displayed peptide co-expressed with phage coat protein pIII) was recognized in all seven sequenced phage clones by mAb 8F12 but not in the M13 plain phage (Figure 3A). Positive phage clone 8F7 (as a representative) could be recognized and pulled down by mAb 8F12 at both non-denatured and denatured conditions through immunoprecipitation (Figure 3B).

### Sequence analysis and alignment

Single stranded phage DNAs extracted from the ten positive clones were sequenced and amino acid sequences of these inserted phage peptides were deduced, as shown in Table 1. Alignment with the original sequence of *Ts*-Pmy revealed that the positive phage clones 8F6, 8F7, 8F10 and 8FJJ had the best matches with region 6–20 aa of *Ts*-Pmy with 4 consensus amino acid residues and most serines conserved, while clones 8A1, 8F1 and 8A9 shared 3 or 2 consensus amino acid residues with the same region (Table 1). The sequence alignment results are consistent with the B cell epitope within *Ts*-Pmy 4-23 aa predicted by the BepiPred program (http://www.imtech.res.in/raghava/abcpred/index.html). The peptides displayed by these positive phage clones might be conformational epitopes of parental antigen *Ts*-Pmy-N from which the mAb 8F12 was induced. The B cell epitope recognized by mAb 8F12 is possibly located between 6-20aa of *Ts*-Pmy. 

**Table 1:** Alignment of the displayed peptide sequences of positive phage clones with original sequence of *Ts*-Pmy.

Clone	Amino acid sequence of insert																						
8F1				L	P	W	H	F	K	***S***	R	H	***R***	Y	Q								
8A9					E	W	M	***S***	H	G	H	P	***R***	P	N	N							
8F10						***S***	L	***S***	T	P	A	T	***R***	H	F	***S***	G						
*Ts*-Pmy (1-23aa)	M	S	L	Y	R	***S***	P	***S***	***A***	***S***	***V***	M	***R***	***S***	A	***S***	M	***L***	***S***	***R***	S	G	G
8JJ^1^								***S***	V	***S***	***V***	G	M	K	P	***S***	P	R	P				
8F7									L	***S***	T	P	Y	***S***	K	***S***	Q	A	***S***	T			
8F6										***S***	H	W	N	***S***	H	***S***	T	P	A	***R***	A		
8A1									***A***	L	S	T	P	T	F	***S***	T	***L***	P	A			

Four phage clones (8F6, 8F7, 8F10 and 8JJ) had 4 consensus amino acid residues with region 6–20 aa of *Ts*-Pmy, while three other clones shared 3 or 2 consensus amino acid residues with the same region (Table 1). The amino acids identical to *Ts*-Pmy (6-20 aa) were highlighted.

1 The peptide sequence of clone 8A6, 8A7, 8A11 and 8A12 were identical and renamed as 8JJ.

**Figure 4:**
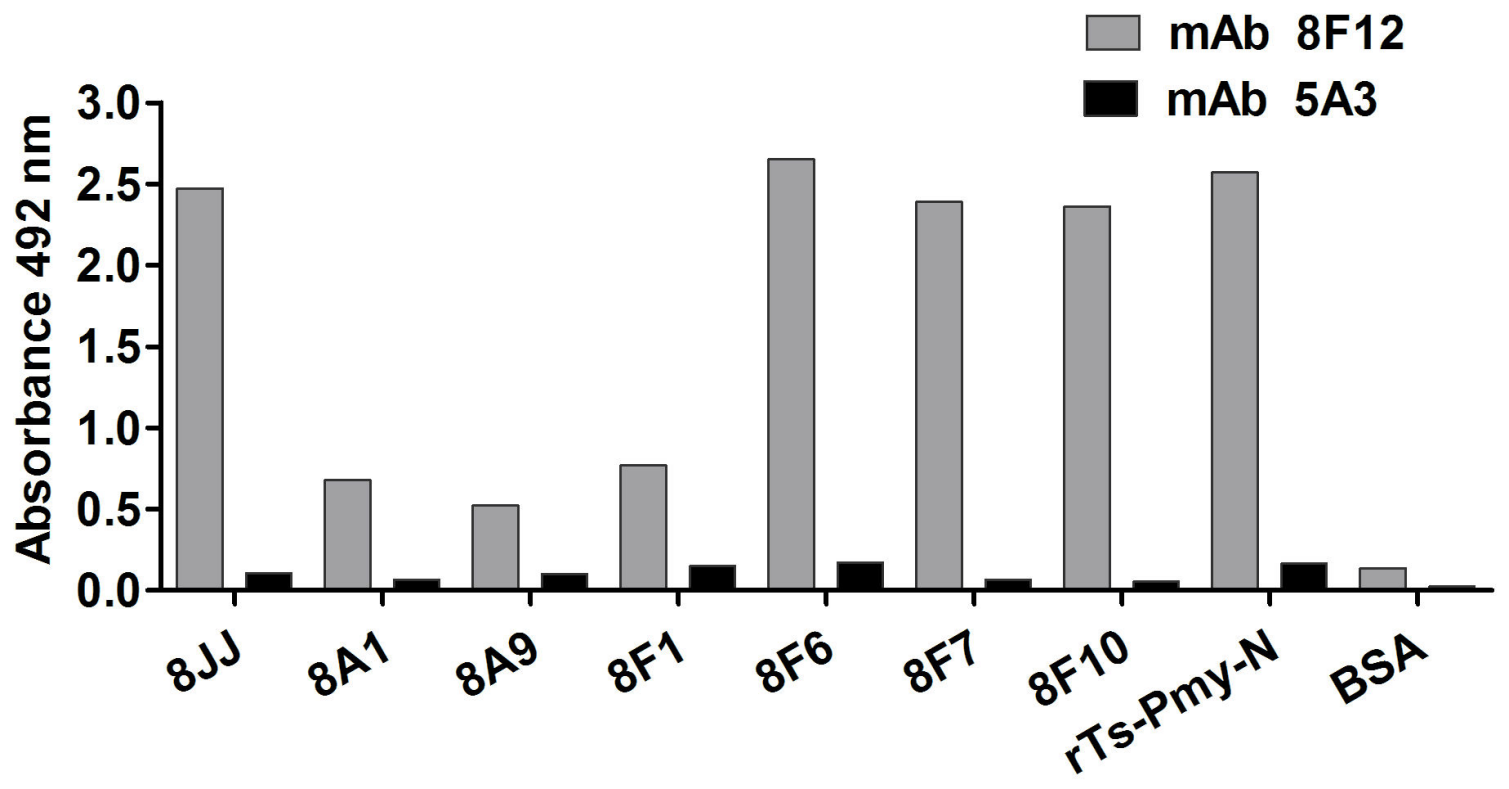
Specific binding of the synthesized peptides to mAb 8F12 as shown by ELISA. These results showed that synthesized peptides conjugated to BSA bound specifically to mAb 8F12. The irrelevant mAb 5A3 was used as a negative control and r*Ts*-Pmy-N served as a positive control. BSA-coated wells were used to exclude non-specific binding.

The peptides of these seven positive phage clones were synthesized and conjugated to BSA or KLH. An ELISA of BSA-conjugated peptides demonstrated that all of them were recognized by mAb 8F12, especially the peptides 8JJ (8A6, 7, 11, 12), 8F6, 8F7 and 8F10, which shared a similar antigenicity with r*Ts*-Pmy-N. There was no peptide reaction with the irrelevant mAb 5A3 (Figure 4). 

### Protective immunity elicited by individual-epitope peptides

**Figure 5:**
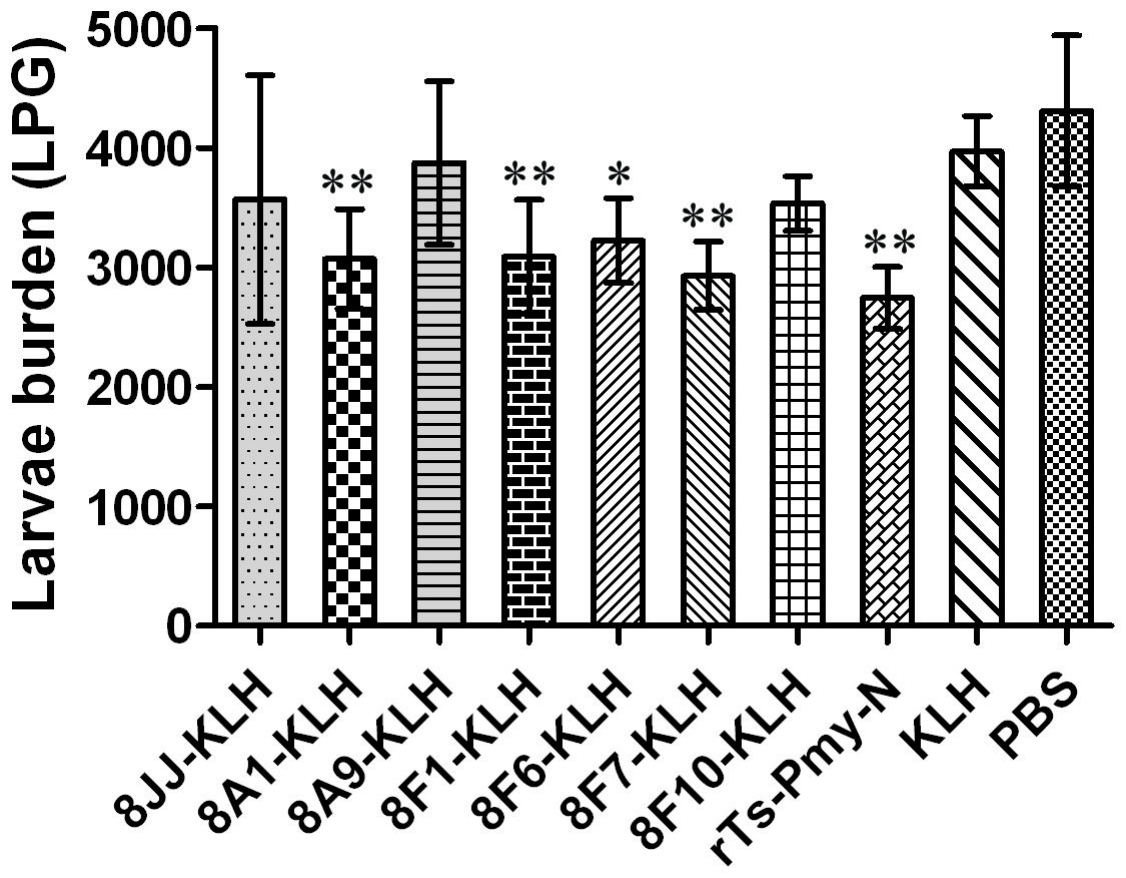
Protective immunity induced by individual epitope peptides conjugated to KLH in vaccinated mice. When compared to the KLH control group, mice immunized with 8A1-KLH, 8F1-KLH, 8F6-KLH and 8F7-KLH experienced 22.7%, 22.2%, 18.7% and 26.3% reduction in the muscle larvae burden, respectively, with a significant difference in comparison to the KLH control (**p < 0.01, *p < 0.05). The larvae per gram (LPG) of muscle shown for each group represent the mean value from 6 animals with the standard deviation (one representative experiment of three independent experiments).

Mice immunized with KLH-conjugated peptides 8A1, 8F1 and 8F7 and subsequently challenged with *T. spiralis* larvae resulted in a 22.7%, 22.2% and 26.2% reduction in muscle larvae burden, respectively. These reductions were statistically significant compared to the KLH control (*p* < 0.01), which was slightly lower than that induced by immunization with r*Ts*-Pmy-N (30.9% reduction in muscle larvae burden). Mice immunized with 8F6-KLH had only 18.8% reduction in muscle larvae burden compared to the KLH control (*p* < 0.05, Figure 5).

### Protective immunity elicited by multi-epitope vaccine

**Figure 6:**
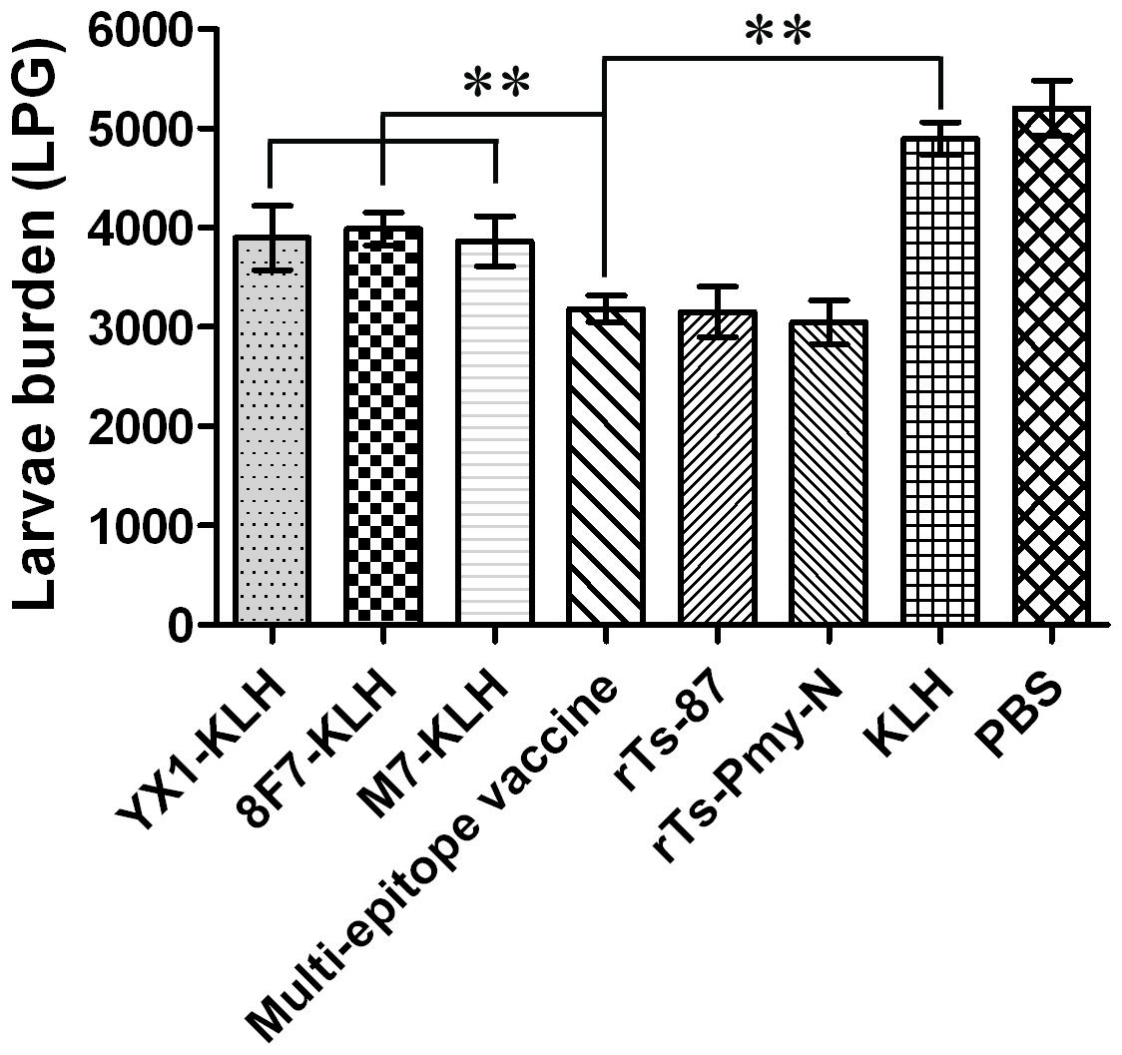
Protective immunity against *T. spiralis* challenge induced by multi-epitope vaccine in vaccinated mice. When compared to the KLH group, mice immunized with multi-epitope vaccine (YX1-KLH, 8F7-KLH and M7-KLH mixture) experienced a 35.0% reduction in muscle larvae burden (**p < 0.01), which was similar to those induced by r*Ts*-87 (35.6%) and r*Ts*-Pmy-N (37.8%), but significantly higher (**p < 0.01) than those induced by an individual peptide (YX1-KLH 20.4%; 8F7-KLH 18.6% and M7-KLH 21.1%). The larvae per gram (LPG) of muscle shown for each group represent the mean value from 6 animals with the standard deviation (one representative experiment of three independent experiments).

Peptide 8F7 induced the highest protection in this study. It was combined with YX1, another epitope peptide of *Ts*-Pmy [29], and *Ts*-87 epitope peptide M7 [28] to formulate a multi-epitope vaccine. The protective immunity induced against *T. spiralis* infection by multi-epitope vaccination was evaluated in the mouse challenge model and compared to the individual peptide immunization. In comparison to the KLH group, mice immunized with multi-epitope vaccine had a 35.0% reduction in muscle larvae burden, which was similar to those induced by r*Ts*-87 (35.6%) and r*Ts*-Pmy-N (37.8%), but significant higher (*p* < 0.01) than those induced by an individual peptide (YX1-KLH 20.4%; 8F7-KLH 18.6% and M7-KLH 21.1%, Figure 6), even if the amount of each peptide in the multi-epitope mixture (17 µg) was only one-third of the amount in the individual peptide group (50 µg). These results showed that the multi-epitope vaccine induced better protective immunity than the individual epitope vaccine against *T. spiralis* infection in BALB/c mice, at the similar level induced by the recombinant *Ts*-Pmy-N formulated with the same adjuvant ISA 50V2.

### Antibody assay

**Figure 7:**
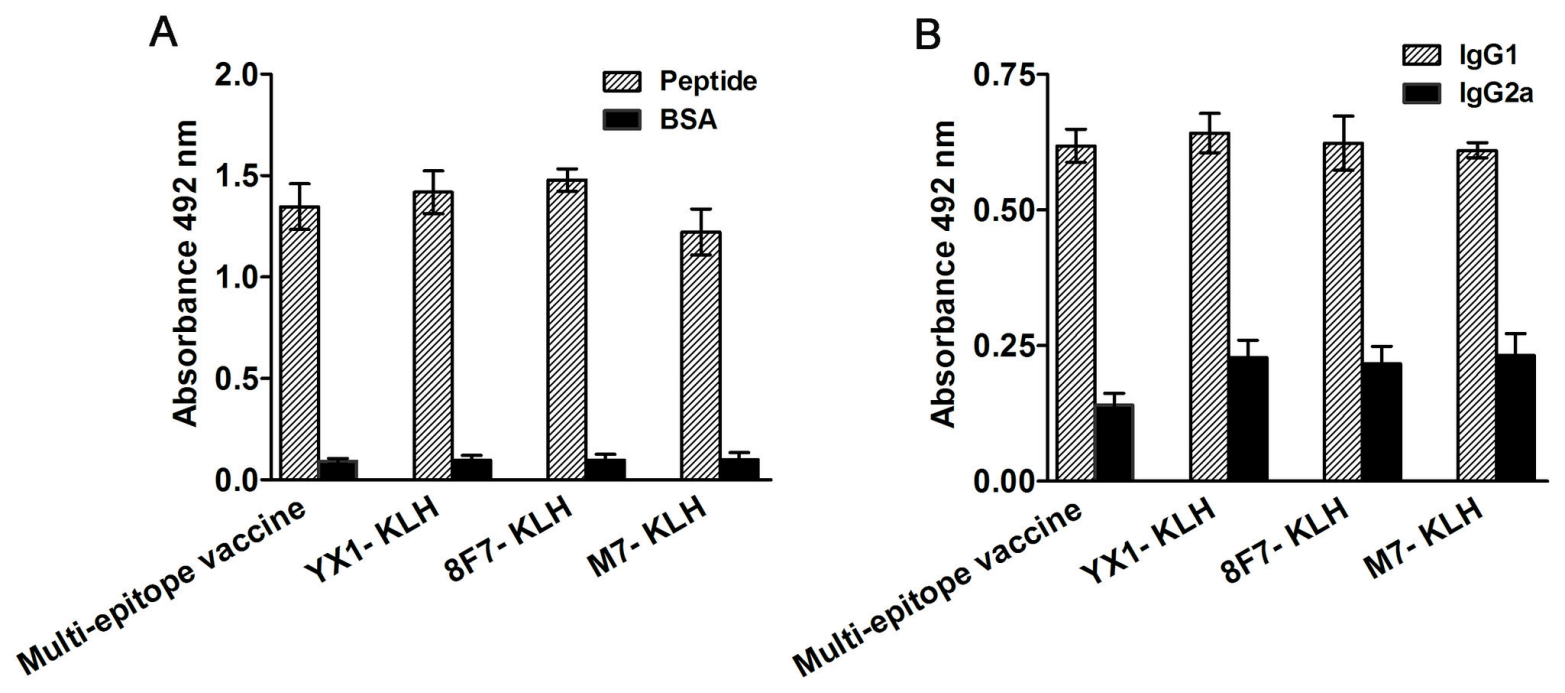
Total specific IgG (A) and IgG1/IgG2a (B) levels in sera of mice immunized with multi-epitope vaccine or individual-epitope peptides detected by ELISA. Individual BSA-conjugated peptide or a mixture of three BSA-conjugated peptides in a total amount of 1 µg was coated on each well of a 96-well plate. HRP-conjugated anti-mouse IgG, IgG1 or IgG2a were used as secondary antibody. The values shown for each group represent the mean OD_492_ value from 6 animals with the standard deviation.

All mice vaccinated with multi-epitope vaccine or individual peptide YX1, 8F7 and M7 conjugated to KLH produced high levels of specific IgG against parental antigen peptide(s) after the third immunization (Figure 7A). Higher IgG1 levels than IgG2a were observed in all vaccinated mouse groups (Figure 7B), suggesting a Th2 predominant immune response in the peptide vaccinations. Mice immunized with PBS or KLH did not show any IgG or IgG isotype responses against the peptide (data not shown).

## Discussion

Epitope vaccines have developed rapidly in recent years [34], with many promising results and substantial protections being reported against viral, bacterial or even parasitic infections [35-38]. A number of epitope vaccines have been tested on humans in clinical trials [39,40]. Epitope vaccines have many advantages compared to the whole protein antigen, including increased safety, rational engineering of epitopes for increased potency and breadth, and the ability to focus the immune response on conserved epitopes [41]. Epitope-based vaccines not only focus the immune response on the effective protective motif of the target antigen, but they also allow for the construction of a chimeric vaccine by engineering multiple effective epitopes from different candidate antigens, thereby improving the level of protection in addition to granting higher safety and avoiding immune evasion or competition from whole antigens. Due to the complexity of the *T. spiralis* life cycle and diversity of its antigens, it is difficult to obtain effective immune protection with a single antigen. A multi-epitope vaccine will be a novel approach for developing more effective vaccines against trichinellosis. 

It has been demonstrated that antibody-independent mechanisms were involved in the expulsion of gastrointestinal nematodes. T helper type 2 (Th2) responses are important in protective immunity against helminth infections [42,43]. For the intestinal dwelling nematode *T. spiralis*, protective immunity is associated with the activation of Th2-type cells within the mesenteric lymph node (MLN) [44]. Early elevations in the Th2 response might be associated with the innate immune responses of intestinal epithelial cells against *T. spiralis* larval invasion [45]. IL-4R alpha-deficiency mice exhibited substantially reduced parasite expulsion, intestinal pathology and Th2 responses [42]. Our previous study also showed that mice immunized with vaccine antigens r*Ts*-Pmy and r*Ts*-87 exhibited predominant Th2-type responses associated with partial protection against *T. spiralis* infection [20,21]. Therefore, it is feasible and practical to identify immuneprotective B cell epitopes within vaccine candidate antigens to develop an epitope vaccine against *T. spiralis*.

An efficient way of identifying protective B cell epitope(s) in a vaccine antigen is to produce a neutralizing or protective mAb against it, and then determine the epitope(s) recognized by mAb [46]. Phage display random peptide library screening has become a key methodology for discovering and mapping antibody epitopes [47]. Not only the linear epitopes but also the conformational epitopes may be identified in this fashion. Many antigenic epitopes have been identified with this approach [48-50]. 

To identify the protective epitope of *Ts*-Pmy, a vaccine antigen of *T. spiralis*, the specific mAb 8F12 was produced against the N-terminus of *Ts*-Pmy. The IgG1 isotype of this antibody indicates a Th2-based response [51]. The passive transfer of this monoclonal antibody to naive mice triggered significant protection against a *T. spiralis* larval challenge. Seven distinct B-cell epitopes of *Ts*-Pmy were identified by immunoscreening a random phage display peptide library with mAb 8F12. The peptide sequences of these identified epitopes shared only 3-4 consensus amino acids with the region of original *Ts*-Pmy-N between 6-20aa, with most serines conversed (Table 1), indicating that they are not the linear, but the conformational epitopes of *Ts*-Pmy-N. It is consistent with the prediction of B cell epitope of *Ts*-Pmy within 4-23aa by BepiPred program. In order to affirm if the peptide displayed by positive phage 8F7 used in this multi-epitope vaccine study is conformationaly related, an additional immunoprecipitation study with anti-*Ts*-Pmy mAb was done. At non-denatured condition, the positive phage 8F7 was specifically pulled down by mAb 8F12, indicating the specific mAb recognized phage 8F7 at its native conformation, it is consistent with the ELISA results showing the mAb recognized the native phage 8F7. However, the mAb also recognized the denatured phage in this immunoprecipitation study, similar to the Western blot results with denatured condition. 

The phage display peptide library used for screening the peptides recognized by anti-*Ts*-Pmy mAb is made based on random combination of 12-mer peptide expressed at the N-terminus of a minor coat protein (pIII) of M13 phage, not directly made from *Ts*-Pmy sequence. Therefore, it is not uncommon that antibody recognized 12-mer peptides from peptide library do not completely match the sequence of native antigen as long as they are conformational and functionally mimic the native epitope (called mimotopes) [52,53]. Studies have shown that immunization with the mimotopes of conformational epitopes could also induce protective immunity [49,53]. In this study, the immunization of mice with these selected peptides produced some protection against a *T. spiralis* larval challenge, although the protection level was lower than that induced by recombinant *Ts*-Pmy-N.

To increase effective protection, the epitope 8F7 that induced the highest protection in this study was combined with another two previously identified epitopes, namely, YX1 (*Ts*-Pmy) and M7 (*Ts*-87), to formulate a multi-epitope vaccine. Mice vaccinated with this multi-epitope vaccine had induced significantly higher levels of protection in comparison to the individual peptide vaccinations, even if the amount of each peptide in the multi-epitope mixture was only one-third of the amount in the individual peptide group. This protection is associated with a high level of total IgG (Figure 7A). The predominant IgG1 subclass observed in the vaccinated mice suggests a Th2 type of immune response in relation to the protection (Figure 7B). These results demonstrate that the multi-epitope vaccine induced better protective immunity against *T. spiralis* infection than an individual-epitope vaccine did, and less peptide was used. However, the overall protection is still low (35% of muscle larva reduction) and exact protective mechanism induced by the multi-epitope vaccine is under investigation. It is a very common dilemma we face for most helminth vaccine developments with low worm reduction (30-50%) or non-sterilizing immunity induced by single or multiple subunit vaccine [54,55], most possibly due to the complexity of the life cycle and multicellular constitution of helminthic parasites and the sophisticated mechanism that parasites develop to evade host immune response. Although the disease development by helminthic parasites usually depends on the intensity of infection [54] and low infection of *T. spiralis* usually causes asymptomatic [56,57], it is still not acceptable if a non-sterilizing vaccine is used on pig or other domestic animals for meat consumption. Even lower infection of pig is still a source of infection for human. In this context sterilizing immunity should be sought in order to warrant pork safety. In order to increase the vaccine efficacy, a multivalent vaccine targeting different stages of helminthes is necessary. In this study, we developed a three-epitope vaccine targeting two vaccine antigens expressed on both larva and adult stages of *T. spiralis* and induced better protective immunity than an individual epitope. A chimera subunit vaccine with these three protective epitopes arrayed according to their structural characterization, instead of the physical combination of individual peptides, may improve the vaccine's protective efficacy. We are also trying to identify T helper (Th) epitope(s) of *Ts*-Pmy to form a chimeric Th-B epitope vaccine that may increase the protection level. Since mucosal immunity may be important to defense *Trichinella* infection as a first barrier and our previous study with another vaccine antigen Ts87 delivered by attenuated *Salmonella typhimurium* produced long-lasting mucosal and systemic immunity against *T. spiralis* larval infection [7,58], we would also like to use the same strategy to orally deliver the multi-epitope vaccine through attenuated *S. typhimurium* in an effort to enhance local immunity to increase the vaccine efficacy. The serological antibody titer will be monitored upon each immunization to understand the dynamic immune response to the inoculated vaccine. The present study provides useful information for further development of a powerful multi-epitope vaccine against trichinellosis.
